# The Affective Slider: A Digital Self-Assessment Scale for the Measurement of Human Emotions

**DOI:** 10.1371/journal.pone.0148037

**Published:** 2016-02-05

**Authors:** Alberto Betella, Paul F. M. J. Verschure

**Affiliations:** 1 SPECS Lab, N-RAS, DTIC, Universitat Pompeu Fabra, Barcelona, Spain; 2 Institució Catalana de Recerca i Estudis Avançats, Barcelona, Spain; University of Vienna, School of Psychology, AUSTRIA

## Abstract

Self-assessment methods are broadly employed in emotion research for the collection of subjective affective ratings. The Self-Assessment Manikin (SAM), a pictorial scale developed in the eighties for the measurement of pleasure, arousal, and dominance, is still among the most popular self-reporting tools, despite having been conceived upon design principles which are today obsolete. By leveraging on state-of-the-art user interfaces and metacommunicative pictorial representations, we developed the Affective Slider (AS), a digital self-reporting tool composed of two slider controls for the quick assessment of pleasure and arousal. To empirically validate the AS, we conducted a systematic comparison between AS and SAM in a task involving the emotional assessment of a series of images taken from the International Affective Picture System (IAPS), a database composed of pictures representing a wide range of semantic categories often used as a benchmark in psychological studies. Our results show that the AS is equivalent to SAM in the self-assessment of pleasure and arousal, with two added advantages: the AS does not require written instructions and it can be easily reproduced in latest-generation digital devices, including smartphones and tablets. Moreover, we compared new and normative IAPS ratings and found a general drop in reported arousal of pictorial stimuli. Not only do our results demonstrate that legacy scales for the self-report of affect can be replaced with new measurement tools developed in accordance to modern design principles, but also that standardized sets of stimuli which are widely adopted in research on human emotion are not as effective as they were in the past due to a general desensitization towards highly arousing content.

## Introduction

Psychological research on emotions has a long past dating back to the second half of the 19^th^ century with Charles Darwin who explained affective states as means of communication to (ultimately) survive [1] and the, so called, James-Lange theory, which defined the manifestation of emotions as a consequence of physiological changes in arousal [2]. Nonetheless, despite the increasing adoption of psychophysiological measures for the inference of human affect, the field still relies extensively on self-reporting tools. Physiological data, in fact, are prone to artifacts and can present drawbacks, in particular when acquired in ecologically-valid conditions [3–5]. For this reason, a common practice is to couple such measures to self-assessment scales or questionnaires.

**Fig 1:**
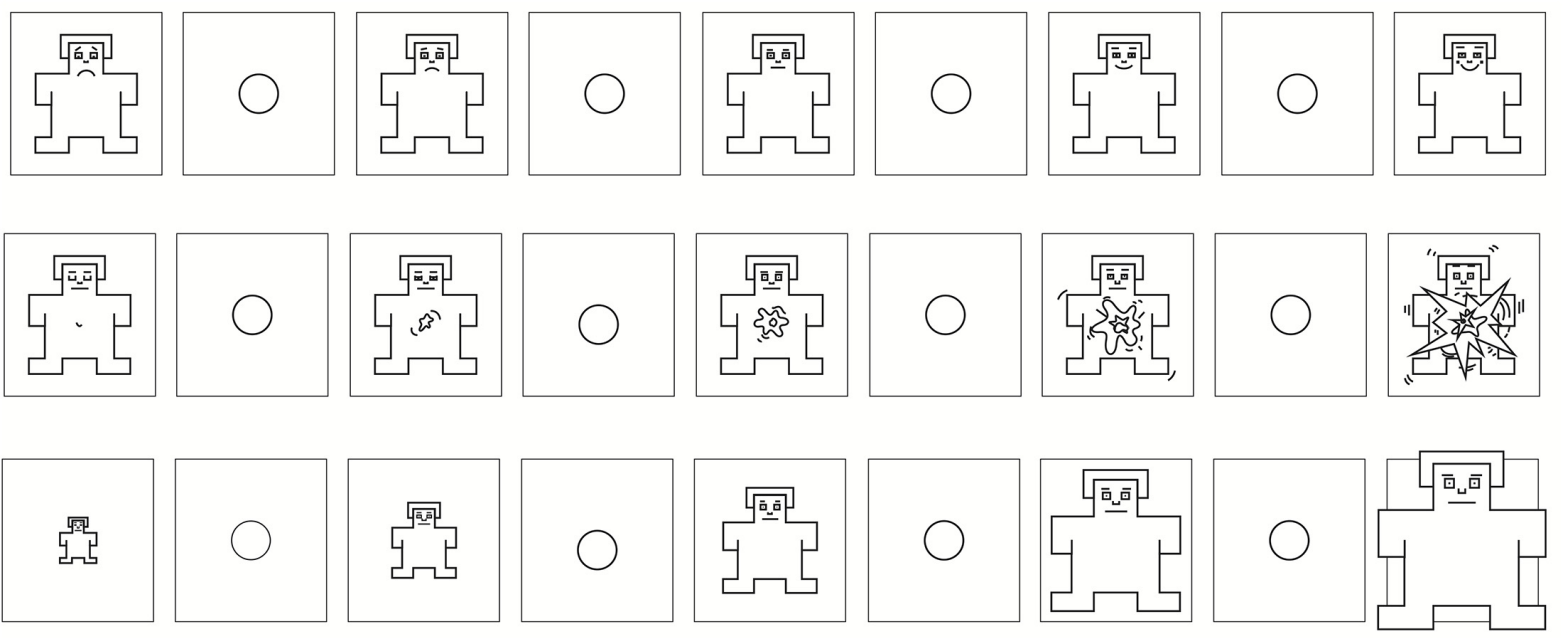
The Self-Assessment Manikin (SAM), adapted with permission from Bradley and Lang 1994 [6]. SAM is a pictorial tool designed in the eighties that measures pleasure (top), arousal (middle) and dominance (bottom) on a discrete scale. It is available in two main versions: paper-and-pencil (5-, 7-, 9-points) and computer program (20-points). Participants can rate their affective state by placing an X over or between any figure.

One of the most popular among the existing self-reporting tools is the Self-Assessment Manikin (SAM) proposed by Bradley and Lang in 1994 [6], which is broadly adopted in psychological studies as well as in a wide range of fields that span from marketing to advertising. SAM is a scale that measures the dimensions of pleasure, arousal and dominance (also called “PAD”) using a series of graphic abstract characters horizontally arranged according to a 9-points scale (even though 5-, 7-points and other variants exist). Pleasure ranges from a frowning to a smiling figure, arousal spans from a sleepy to a widely awake figure showing an incremental explosion at the center, while dominance ranges from a very small to a very large character (Fig 1). The original SAM paper [6] has collected in total over 3200 citations since its publication and it was cited in more than 2200 peer-reviewed scientific articles in the last 5 years alone (source: Google Scholar, http://scholar.google.com).

We have regularly adopted SAM at our laboratory (generally paired with behavioral and phsychophysiological measures) to conduct studies on human emotion because it was the best established tool available for the quick collection of self-reported affective data. Although our participants were administered the official rating instructions, they frequently asked to the experimenter to further clarify the meaning of the pictographic representations, hence raising a pragmatic concern about the intuitiveness of SAM. It should not be surprising indeed that a scale which was designed more than two decades ago might not be understood today as intuitively as it was in the past.

As a matter of fact, the paper-and-pencil design principles upon which SAM was based are distant from the theories that underlie graphical user interfaces at the present-day. We live in an era where advanced interfaces, digital media, social networks and mobile applications changed the way people communicate and, in a broader sense, shaped new paradigms of interaction [7]. By exploiting contemporary design standards for user interfaces along with modern metacommunicative graphical representations of emotions, it is possible to develop novel and more effective tools for psychological research.

**Fig 2:**
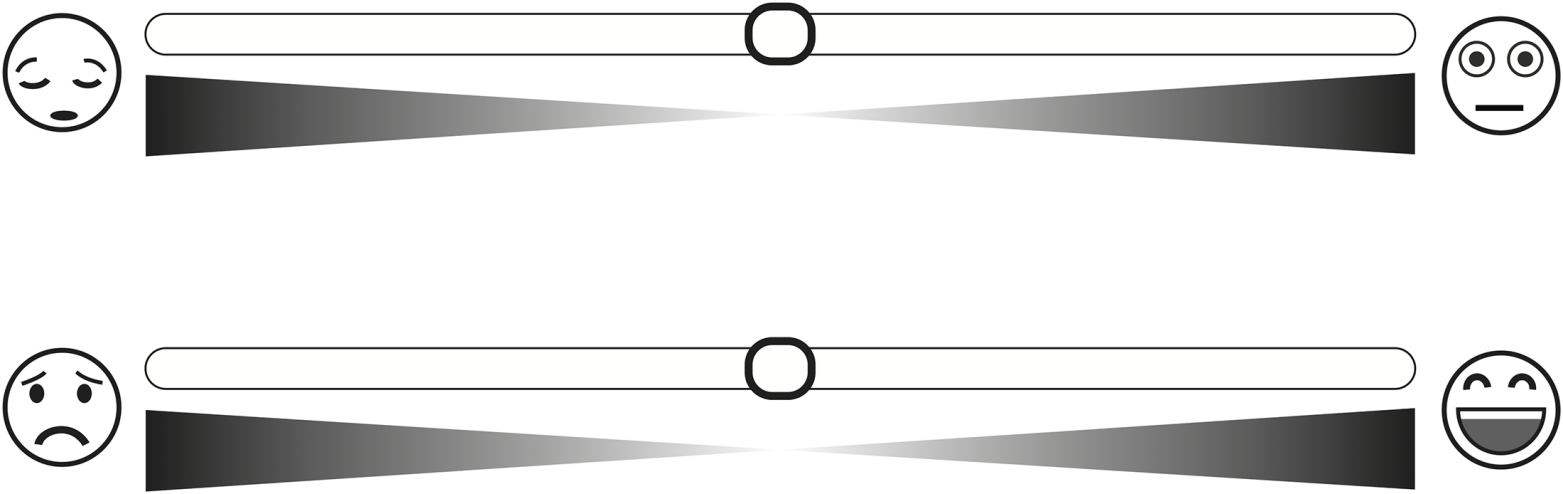
The “Affective Slider” (AS) is a digital self-reporting tool composed of two sliders that measure arousal (top) and pleasure (bottom) on a continuous scale. The AS does not require written instructions and it is intentionally displayed using a neutral chromatic palette to avoid bias in ratings due to the emotional connotation of colors. See text for more details.

For this reason, we designed a new digital scale for the self-assessment of emotion that we called the “Affective Slider” (AS) (Fig 2). The AS is composed of two slider controls that measure basic emotions in terms of pleasure and arousal on a continuous scale that we have systematically calibrated to the SAM in a number of experiments which involved emotional ratings.

Interface elements such as (physical or digital) slider controls have been sparsely adopted in psychological research in tasks related to affective assessment, including video corpora annotation [8] and affective measurements [9, 10]. Yet, the use of sliders in past studies was mainly grounded in intuition. When designing a novel research tool, it is crucial to conduct an empirical validation which allows to compare new methods to their state-of-the-art counterparts. This is precisely what we did with the AS.

Here, we present the results of an experiment which consisted of a systematic comparison between AS and SAM ratings collected through a task that involved the emotional assessment of a series of images taken from the International Affective Picture System (IAPS), a standardized database containing pictures representing a wide range of semantic categories, which had been previously calibrated using the PAD affective dimensions measured through the SAM [11].

The experiment we conducted also allowed us to replicate the original IAPS study through the collection of present-day SAM ratings that we compared to the previous IAPS norms. By doing so, we aimed to validate our hypothesis that the stimuli from this collection (most of which are dated back to the end of the eighties [12]) don’t trigger today the same affective responses as they did in the past. Our society, in fact, is increasingly stimulated with highly arousing media content through the massive exposure to media. This plausibly leads to a desensitization towards such content which has been also highlighted in a number of earlier studies [13–15]. Our assumptions are grounded in previous research that observed general trends of lower arousal associated to the IAPS pictures when compared to their normative ratings [16, 17] without, however, drawing definitive conclusions on this specific outcome.

The current study provides a relevant contribution to the field of emotion research by raising new challenges for current methodologies and by introducing a novel tool for the measurement of affect.

## Materials and Methods

### The Affective Slider

The “Affective Slider” (AS) is a digital scale for the self-assessment of emotion composed of two separate slider controls (or “sliders”) that measure pleasure and arousal.

In the AS, the two independent controls are located one on top of the other (in random order over subsequent trials).

Underneath each slider two isosceles triangles are placed (symmetrically mirrored from the topmost vertex) that serve as a visual cue for intensity.

Stylized facial expressions (also called “emoticons”) visually representing bipolar affective states from Mehrabian and Russell’s emotionality scales (i.e. unhappy/happy for pleasure and sleepy/wide-awake for arousal) [18] lie at the two ends of each slider. Previous studies demonstrated the existence of universal trends in the attribution of affect to colors [19, 20]. To avoid potential biases in ratings, in the design of the AS we adopted a monochromatic neutral color scheme (Fig 2).

Contrary to SAM, the AS does not measure dominance. We excluded this dimension for two main reasons: first, Russel’s bipolar emotional space constitutes the, so called, “core affect” and is sufficient alone to measure basic emotion [21]. As a result, dominance can be considered redundant and seen as a consequence of core affect. Second, this dimension has not shown consistent effects across studies [16].

The introduction of SAM as a non-verbal pictorial scale for the self-assessment of affect tackled most of the issues typical of pre-SAM verbal scales, which were language-dependent and required complex statistical factorial analyses [6]. However, written instructions are still necessary in order to illustrate how to properly use the SAM tool (SAM instructions normally account for more than 500 English words [11]). In this context, the AS constitutes a further advance since, by leveraging on today’s practical knowledge of user interface elements (e.g. sliders) and metacommunicative pictorial representations (i.e. emoticons), it does not require written instructions.

Another advantage of slider controls is the possibility of collecting ratings on continuous scales that allow for more accurate high-resolution measurements, as opposed to the SAM which records data upon a relatively condensed Likert scale.

Due to the obsolescence of the original SAM software version (first developed in 1987), a few researchers more recently implemented their own digital SAM variants which, however, are currently hard to find and not fully-functional. In contrast, the AS can be easily reproduced in any modern digital device, including smartphones and tablets. Its source code and graphic elements, along with examples of implementation, are publicly available under a Creative Commons license (http://AS.specs-lab.com). Moreover, to facilitate researchers in the development of custom-tailored versions of the AS (e.g. integration into existing software packages), we provide detailed design guidelines and recommendations (S1 Guidelines).

### 0.1 Experimental Protocol

We designed an experiment where 400 volunteers were asked to rate a set of 60 pictures randomly selected from the International Affective Picture System (IAPS) [22].

The aim of our study was to empirically validate the AS by correlating the collected affective dimensions to the corresponding SAM ratings and to systematically compare new and normative IAPS ratings. We expected to find a) a strong correlation between the SAM and the AS scales and b) consistently lower ratings of arousal associated to the IAPS pictures when compared to the normative ratings.

We divided our sample into two groups of equal size and assigned each participant to either the SAM or the AS condition, following a between-subjects design (see Section Sample and data pre-processing for more details). The dependent variables measured were “pleasure” and “arousal”.

Previous research empirically demonstrated that psychological studies which involve the administration of internet-based surveys deliver equivalent results to laboratory settings [23, 24]. For this reason, we developed an on-line questionnaire composed of 4 main sections: a) consent form and demographic data collection, b) instructions, c) pictures rating and d) debriefing.

The protocol of the experiment was approved by the local Ethical Committee “Clinical Research Ethical Committee (CEIC) Parc de Salut Mar” (Barcelona, Spain). After having electronically signed the consent form and answered the demographic questions (i.e. gender, age, nationality and level of education), participants were presented with the instructions. We administered the original IAPS instructions [11] to both the experimental groups, with minor changes due to the different medium (e.g. we modernized the references to the “booklet” for the affective ratings and to the “projector” that displayed the pictures). In addition, for the AS condition we removed all the mentioning of SAM along with its instructions and simply asked participants to “move the sliders to express how you actually feel while watching the picture”.

In the same page, and prior to the confirmation button, we included a notification where the subjects were asked to put their web browser in full-screen mode to maximize the questionnaire resolution and avoid external distractions such as software running in the background. Moreover, we displayed a warning message explaining that repeated dummy ratings (e.g. skipping to the following picture too quickly or without actually interacting with the scales) would be automatically detected by the system, with the consequence of a sudden termination and exclusion from the experiment.

**Fig 3:**
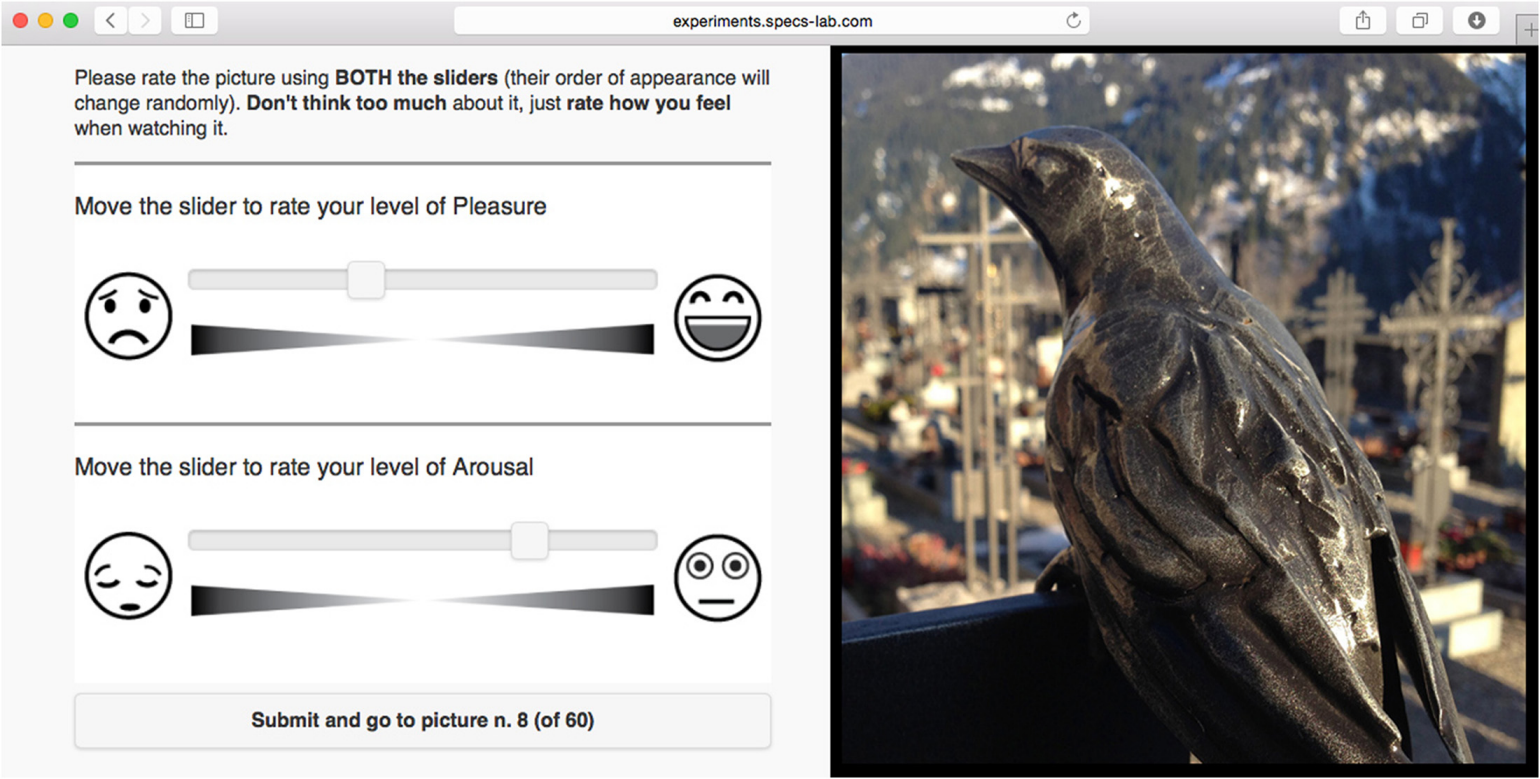
Screenshot of the web-based questionnaire showing a single experimental trial using the AS. The picture (in this example just a placeholder and not part of the IAPS collection) is randomly displayed either on the left or on the right side of the screen. Similarly, the order of the pleasure and arousal dimensions is randomized.

A series of 60 separated pictorial stimuli followed, each of which was sequentially presented in a dedicated page and pseudo-randomly selected (i.e. shuffled) from the entire IAPS collection. Each page was split into two vertical sections that displayed simultaneously the IAPS image on one side, along with the SAM (digital 9-points version we implemented) or the AS on the other (Fig 3).

To prevent potential automaticity in the rating process, the content of these two vertical sections was randomly interchanged for each new trial. Similarly, the order of presentation of the two affective dimensions in the administered scale was randomized (see also the recommendations in S1 Guidelines).

Once the participant completed the rating of one pictorial stimulus, it was possible to proceed to the following trial by pressing the “Submit and Continue” button, until all of the pictures were presented. A 5-seconds black mask covering the entire screen space was displayed between each trial.

Upon completion of all the trials, a debriefing page was presented to the participant, which included the physical and email addresses of the experimenter for further clarifications or questions.

### 0.2 Sample and data pre-processing

400 volunteers were recruited using Amazon’s Mechanical Turk (mturk.com), a popular web service that in recent years has been increasingly employed in the conduction of experiments in psychology and social sciences. Previous research demonstrated that the experimental data collected via Mechanical Turk can be considered as reliable as those obtained via traditional methods [25]. Participants were equally assigned to one of the two experimental conditions (AS or SAM) and they all received a monetary compensation.

Although on-line recruitment and internet-based questionnaires present several advantages, web technologies in scientific research have a shortcoming: there is no straightforward way to ensure that participants follow precisely the given instructions. To systematically control for possible errors or misconduct, along with the data explicitly collected through the questionnaire, we logged into our database the time-stamp of each of the 60 trials associated to the participant’s unique ID (which was also locally stored using a “cookie”), as well as some anonymous information including (partially masked) IP address and browser inner-window resolution of the client.

Indeed, after conducting a preliminary analysis of the dataset aggregated from the entire sample, we observed that 22% of the volunteers did not successfully complete the questionnaire, submitted more than 60 ratings or participated in the experiment twice (probably due to a manual refresh of the web page or to an incorrect use of the “back” button of the browser). Moreover, from the analysis of the browser window size, we found volunteers that used resolutions below 800x500 pixels (i.e. the minimum size to ensure that the pages of our questionnaire were properly displayed).

For these reasons, and to avoid any inconsistency in our data, we automatically excluded all those participants that did not meet the original criteria, either due to inadvertence or negligence, resulting in a final sample of 309 participants (110 females, mean age 36.26±11.15SD), 87% of which held a university or master degree and whose nationality was mainly American (42.4%) and Indian (53.7%).

Among the 309 valid participants, 153 were exposed to the SAM experimental condition (47 females, mean age 36.3±10.7SD), while 156 to the AS condition (63 females, mean age 36.15±11.6SD).

The mean time for the completion of the entire experiment was 19±9 minutes.

## Results

We collected a total of 18540 single ratings, equally divided between SAM and AS on a picture set composed of 1178 different images from the IAPS database, each of which was rated on average 15.2±3.9 times. We averaged the collected scores, thus obtaining mean values of pleasure and arousal for both the AS and the SAM ratings associated with each of the IAPS stimuli. First, we compared the affective ratings between AS and SAM by looking for correlations between the two scales. Second, we compared the new SAM ratings we collected to the normative SAM ratings provided with IAPS (S1 Data). The statistical analysis included the calculation of Spearman’s correlation coefficients and Wilcoxon rank-based tests due to the non-Gaussianity of the distributions as assessed through Kolmogorov–Smirnov tests with Lilliefors correction.

### 0.3 AS versus SAM

**Fig 4:**
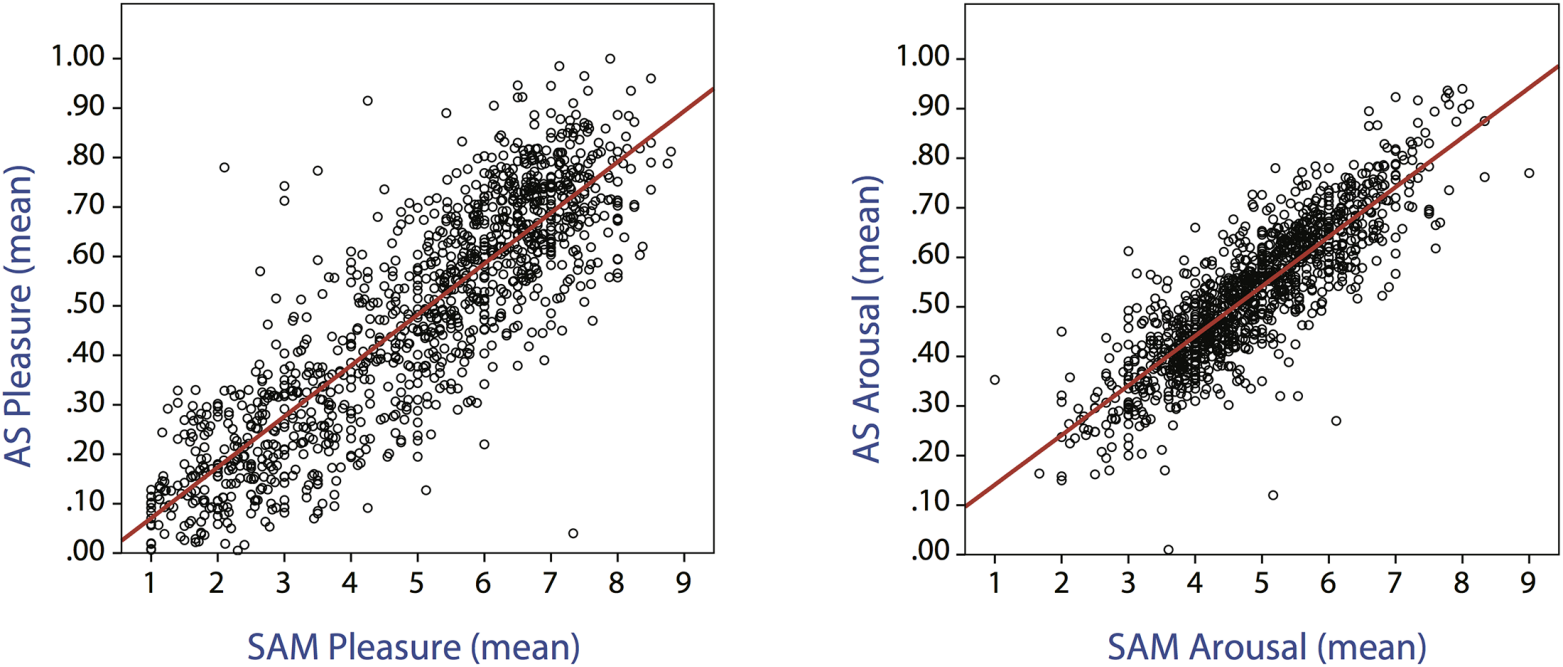
Scatterplots representing the linear correlation between AS and SAM pleasure (r_s_ = .852) and arousal (r_s_ = .860). The red line indicates the best fit.

In order to establish the similarity of the ratings obtained with AS and those rendered through SAM, we calculated the Spearman’s correlation coefficient between the measured affective dimensions. We found a strong correlation between AS and SAM for both pleasure (r_s_(1176) = .852, *p* < .001) and arousal (r_s_(1176) = .860, *p* < .001) (Fig 4)

### 0.4 Comparison between normative and new IAPS ratings

By taking into consideration the experimental group assigned to the SAM condition alone (N = 153), we compared our (“new”) ratings of pleasure and arousal to the (“old”) normative ones provided with the IAPS database. Both old and new ratings were collected using SAM on a 9-points scale and averaged across all subjects. We computed a Spearman’s rank correlation coefficient between old and new ratings of pleasure and obtained a very strong correlation (r_s_(1176) = .9, *p* < .001).

Similarly, we calculated the Spearman’s correlation coefficient between the old and the new ratings of arousal. The results of the test only showed a moderate correlation for the arousal dimension (r_s_(1176) = .58, *p* < .001). To further analyze this outcome, we split the IAPS collection into two equal parts divided by the overall normative median arousal and we called the newly created categories “low arousal” (mean arousal ≤4.8, N = 570) and “high arousal” (mean arousal >4.8, N = 608). For each of these categories, we conducted correlation tests between old and new arousal ratings. We found a moderate correlation for the low arousal category (r_s_(568) = .42, *p* < .001) and a weak correlation for the high arousal category (r_s_(606) = .3, *p* < .001), suggesting that IAPS pictures that were originally categorized as highly arousing were not perceived as such in our study. To follow up these findings, we excluded a subset of pictures associated to central arousal values in the original IAPS dataset (normative Mdn arousal = 4.85, mean arousal = 4.81±1.15) and distributed our data into two more extreme categories that we called “very low arousal” (mean arousal ≤4, N = 313) and “very high arousal” (mean arousal ≥6, N = 204). We submitted both categories to a Spearman’s test to look for correlations between old and new arousal ratings. We found a weak correlation for the very low arousal category (r_s_(311) = .3, *p* < .001), while the very high arousal category did not show any significant correlation between the old and the new arousal ratings with a correlation coefficient roughly equal to zero (r_s_(202) = .02, *p* > .1). We conducted a Wilcoxon Signed-Ranks test for the pictures belonging to the very high arousal category (N = 204) and compared the old ratings of arousal (Mdn arousal = 6.46, mean arousal = 6.48±0.35) to the new ones (Mdn arousal = 5.81, mean arousal = 5.82±1). The results of the test indicated that the new ratings were significantly lower that the old ones (Z = -7.6, *p* < .001).

## Conclusion

Emotion research has made considerable progress in recent years, in particular due to the increasing diffusion of methods for the inference of affect grounded in direct and indirect physiological responses modulated by the Autonomic Nervous System. Because such measures are easily contaminated with artifacts [26, 27] and the state of the art about their interpretation is still growing, it is a common practice to couple them with self-assessment tools which provide a complementary source of information. Although psychological research on emotion is flourishing in terms of novel approaches and materials, some aspects of the field remain anchored to legacy methods. The Self-Assessment Manikin (SAM), for instance, is one of the most diffused self-reporting scales, despite having been designed more than two decades ago. In the past years, a number of experiments we conducted at our laboratory highlighted the need for a more intuitive and modern tool for the self-assessment of affect. This is precisely why we designed the Affective Slider (AS), a non-verbal digital scale which, by using two separate slider controls, allows to collect in real-time self-reported ratings of pleasure and arousal.

In this study, we present the results of an empirical validation where we compared the affective ratings we collected through SAM and AS from a fairly large sample using a series of pictures from the IAPS collection, one of the most popular and widely adopted set of pictorial stimuli covering a broad range of affective semantic categories.

The purpose of our experiment was twofold. On the one hand, our goal was to systematically validate the AS as a reliable scale for the quick measurement of the affective dimensions of pleasure and arousal. On the other hand, we aimed to find whether the normative ratings provided with the original IAPS study from Lang et al. 1999 [28], a benchmark in psychological research, could be replicated today.

Our results show a very strong correlation between the SAM and AS ratings, hence empirically demonstrating that the AS can replace SAM in the self-reporting of pleasure and arousal, with the additional advantages of being self-contained and easily reproducible in latest-generation digital devices.

In contrast to SAM, in fact, the AS exploits new universal skills acquired through the large diffusion of modern electronic devices (e.g. interaction with interface elements such as sliders) and it does not require written instructions thus relying exclusively on non-verbal cues. For these reasons, we will henceforth adopt the AS in tasks that involve the self-reporting of pleasure and arousal. We invite other researchers to do the same and, to facilitate this, we provide detailed guidelines along with the source code and graphic elements to easily reproduce the AS in future studies (see S1 Guidelines).

In addition, grounded in previous research that highlights a general desensitization towards high arousing visual content [13–15], we compared the new IAPS ratings collected through SAM to the normative data.

When taking into account the entire spectrum of IAPS stimuli, we only found a moderate correlation for the arousal dimension between new and old SAM ratings. This result clearly indicates a drop in the population’s sensitivity when exposed to arousing content. Most importantly, by partitioning the set of IAPS stimuli into lower and higher extremes, we found that the new arousal ratings for pictures categorized as highly arousing in IAPS (204 pictures with mean normative arousal ≥6 on a 9-points scale) were significantly lower than and utterly uncorrelated with the normative ones (r_s_ = 0.02).

Previous studies that involved comparisons between more recent and normative IAPS ratings found similar results. Libkuman et al. [16], for instance, obtained lower arousal ratings than the IAPS norms. However, their work mainly aimed to extend a subset of 703 IAPS pictures through the collection of new dimensions and adopted a different protocol that involved the use of 14 different Likert scales rather than SAM. Since these changes might have acted as confounding variables, the authors limit their interpretation of this outcome to a speculation about the factors that might have led to it. Similarly, Grühn and Scheibe [17], while evaluating the impact of age on IAPS ratings using a pictorial set composed of 504 stimuli, found a lower correlation between new and normative arousal ratings.

We have no evidence that the methods we applied in our research might have interfered with the obtained outcome. For the collection of IAPS ratings, we systematically followed a setup that reflected the original experimental settings, including the instructions administered to the participants. Moreover, our results perfectly replicate the normative ratings of pleasure with a very strong correlation (r_s_ = 0.9), thus confirming the coherence of this dimension in the IAPS database across time and consequently implying that the significant effect we found for arousal is genuine.

To the best of our knowledge, our investigation constitutes the most comprehensive and recent study that purposefully replicated the IAPS norms. Our results are in complete agreement with those obtained in previous studies and further extend them to a broader pool of stimuli (i.e. 1178 IAPS pictures). In addition, our findings show that the most arousing IAPS subset consisting of over 200 pictures is perceived today as significantly less arousing, hence suggesting that the use of highly arousing visual content from standardized databases such as IAPS as a benchmark to elicit strong affective responses measured through self-assessment, psychophysiological signals or neuroimaging techniques can lead to inaccurate results, since those stimuli are not calibrated to contemporary sensitivity.

Not only do our results demonstrate that legacy scales for the self-report of affect such as the SAM can be replaced with more intuitive measurement tools developed in accordance to modern design principles, but also that affective responses might combine both invariant and variant components, suggesting a larger plasticity in emotional appraisal and expression than initially expected. This raises the specific question to what extent human emotional experience and expression can be further molded by experience in particular along the dimensions of pleasure and arousal.

Further improvements will consist in the creation of a new set of original pictorial stimuli able to trigger strong responses in arousal. Such database could be implemented using an open web-based collaborative platform (e.g. a Wiki) where researchers can contribute with novel pictures that will be collectively rated using the AS.

## Supporting Information

S1 Guidelines Affective Slider design and implementation guidelines, including the URL to download the source code and graphic elements. (PDF)

S1 Data Normative and new (2015) ratings of 1178 pictures from the IAPS database collected using the Self-Assessment Manikin. (ZIP)
