# Supplementary material for: The Affective Slider: A Digital Self-Assessment Scale for the Measurement of Human Emotions
Source: PLoS One. 2016 Feb 5;11(2):e0148037. doi: 10.1371/journal.pone.0148037 (PMC4743948; doi:10.1371/journal.pone.0148037)
Supplement: S1 Guidelines — (PDF) [file pone.0148037.s001.pdf]

The Affective Slider: a Digital Self-Assessment Scale for the Measurement of Human Emotions

Alberto Betella, Paul F.M.J. Verschure

Supporting Information

S1 Guidelines. Affective slider design and implementation guidelines
The Affective Slider (AS) source code and graphic elements, along with some examples of web-based implementations, are released under a Creative Commons license and publicly available at the web address: http://AS.specs-lab.com.

Custom-tailored implementations of the AS can be developed using any programming framework (e.g. iOS or Android SDK), as long as they fully comply with the following guidelines:

- Both the pleasure and arousal slider controls must have an horizontal orientation and be presented simultaneously with the slider thumbs (i.e. the movable elements) always placed at the center of the tracks. For right-to-left writing cultures the AS can be horizontally mirrored;
- The spacing between the two sliders and their size must be adequate (and proportional to the screen) to allow the user to easily select the desired values;
- Each one of the sliders must be followed by the intensity cue including the associated emoticons placed at the two extremities;
- All the elements composing the AS must adopt exclusively a greyscale color palette. In case the AS is used on a black or dark background, the default color palette should be inverted (i.e. black to white);
- Each slider measures a single value on a continuous normalized scale ranging from 0 to 1 with a central value (i.e. default thumb position) equal to 0.5 and a minimum resolution of 100 steps (i.e. least step = 0.01). This resolution can increase (e.g. least step ≤ 0.001) in case of devices with high-resolution screens, such as desktop PCs and tablets;
- To allow for an easy manipulation, the sliders thumbs must have the shape of a perfect circle or square (with optional rounded corners) and be at least 10% bigger than the track bar height.

Moreover, in order to discourage potential biases in ratings, we strongly suggest to implement the following recommendations:

- The presentation order of the two sliders should be randomized (i.e. pleasure above and arousal below, or vice versa);
- User interactions with the thumbs such as clicks or taps should be detected to provide immediate visual feedback (e.g. upon interaction, the thumb filling color changes from white to black) and to identify potential dummy ratings (e.g. to prevent the user to continue until a rating is performed or to mark the data point as non-genuine for posterior exclusion in case the thumb is not tapped at all).
